# Biofilm-mediated infections by multidrug-resistant microbes: a comprehensive exploration and forward perspectives

**DOI:** 10.1007/s00203-023-03826-z

**Published:** 2024-02-14

**Authors:** Mai M. Zafer, Gamal A. Mohamed, Sabrin R. M. Ibrahim, Soumya Ghosh, Charné Bornman, Mahmoud A. Elfaky

**Affiliations:** 1 Department of Microbiology and Immunology, Faculty of Pharmacy, Ahram Canadian University, Cairo, Egypt; 2 Department of Natural Products and Alternative Medicine, Faculty of Pharmacy, King Abdulaziz University, 21589 Jeddah, Saudi Arabia; 3 Department of Chemistry, Preparatory Year Program, Batterjee Medical College, 21442 Jeddah, Saudi Arabia; 4 Department of Pharmacognosy, Faculty of Pharmacy, Assiut University, Assiut, 71526 Egypt; 5 Department of Engineering Sciences, Faculty of Natural and Agricultural Sciences, University of the Free State, Bloemfontein, 9301 South Africa; 6 Center for Artificial Intelligence in Precision Medicine, King Abdulaziz University, 21589 Jeddah, Saudi Arabia; 7 Natural and Medical Science Research Center, University of Nizwa, Nizwa, 616 Oman

**Keywords:** Biofilm, Antibiotic resistance, Chronic infections, Antibiofilm strategies, Future perspectives

## Abstract

A biofilm is a collection of microorganisms organized in a matrix of extracellular polymeric material. Biofilms consist of microbial cells that attach to both surfaces and each other, whether they are living or non-living. These microbial biofilms can lead to hospital-acquired infections and are generally detrimental. They possess the ability to resist the human immune system and antibiotics. The National Institute of Health (NIH) states that biofilm formation is associated with 65% of all microbial illnesses and 80% of chronic illnesses. Additionally, non-device-related microbial biofilm infections include conditions like cystic fibrosis, otitis media, infective endocarditis, and chronic inflammatory disorders. This review aims to provide an overview of research on chronic infections caused by microbial biofilms, methods used for biofilm detection, recent approaches to combat biofilms, and future perspectives, including the development of innovative antimicrobial strategies such as antimicrobial peptides, bacteriophages, and agents that disrupt biofilms.

## Introduction

Biofilms are known to be the prevalent form of microbial life, with most of the microbes living as biofilm communities in diverse surroundings, within host organisms (Flemming et al. 2016). Microbes display two kinds of growth: free-living planktonic; surface attached within biofilms, which are structured communities enclosed in a self-produced or extracellular polymeric matrix composed of water and extracellular polymeric substances (EPS), primarily polysaccharides, proteins and DNA (Rumbaugh and Sauer 2020). Biofilm formation begins when planktonic microorganisms attach to surfaces, marking a crucial stage in the transition of individual microorganisms into a structured community. This adherence process initiates the development of the microorganisms from their free-floating state into a cohesive and organized biofilm structure (Haggag 2010). In the early phase of biofilm formation, microorganisms attach to surfaces in a loosely bound and reversible manner. This stage is characterized by the presence of microorganisms attached to surfaces in a polar manner. Subsequently, the microorganisms reorient themselves to lie flat on the surfaces, establishing irreversible attachment. This irreversible attachment leads to the development of resistance against various physical factors that could otherwise impede the formation of the biofilm (Banerjee et al. 2015). Following the successful attachment of microorganisms to surfaces, the adhered microorganisms initiate multiplication and aggregation within the extracellular polymeric substance (EPS) they produce. This process leads to the formation of microcolonies, especially in the presence of a high concentration of c-di-GMP. Interactions between microorganisms and surfaces rely on the movements facilitated by flagella and type IV pili-mediated motilities. Flagella are crucial for the microorganisms' interactions with surfaces, while type IV pili play a significant role in cell-to-cell aggregations, facilitating the formation of microcolonies (Rabin et al. 2015). The extracellular polymeric substance (EPS) plays a vital role in the maturation of biofilms by fulfilling several functions. It aids in the attachment of microbes to surfaces, providing stability to the three-dimensional structure of the biofilm. Additionally, EPS facilitates the clustering of cells together, offering protection against various stresses such as the immune system response of the host, antimicrobial agents, oxidative damage, and metallic cations. Furthermore, EPS serves as a protective capsule, encapsulating signalling molecules necessary for quorum sensing, as well as metabolic products and enzymes essential for biofilm function (Toyofuku et al. 2016). Ultimately, mature biofilms undergo dispersion, either actively through mechanisms involving motility and degradation of the extracellular polymeric substance (EPS), or passively through physical factors such as liquid flow. This dispersion process allows the microorganisms within the biofilm to disperse and initiate a new cycle of biofilm formation elsewhere. Several factors contribute to the dispersion of mature biofilms, including an overgrown population, intense competition among microorganisms, and a scarcity of nutrients. These factors primarily drive the dispersal of the mature biofilm, facilitating the colonization of new surfaces and the initiation of biofilm formation (Rabin et al. 2015) Biofilm formation is a significant virulence mechanism and a hallmark feature of problematic pathogens that are multidrug resistant (MDR) in hospital settings (Roy et al. 2022). Microorganisms that can form complex structures called biofilms can colonize biotic and abiotic surfaces for prolonged periods of time, and can grow and replicate even under unfavourable conditions (Eze et al. 2018). These organisms could persist in adverse environmental conditions such as hospital settings where there is excessive use of antibiotics and disinfectants (Roy et al. 2022). They also have diverse tools to facilitate and maintain the formation of biofilms in the hospital environment. The development of biofilm is a highly regulated process that is due to the relative contributions and interactions of genetics (active response) and environmental conditions (passive response) (Bjarnsholt et al. 2018).

Chronic infections caused by multidrug-resistant (MDR) pathogens present a great challenge for eradication due to their resistance to conventional antibiotics, as well as their ability to form biofilms and persistance over time. Furthermore, these infections can also influence the host's immune response. (Singh et al. 2022). Persistent biofilm-related infections pose a clinical threat in terms of the morbidity and mortality rates of patients and healthcare-associated costs (Assefa and Amare 2022). Microbial biofilms in hospital settings can be produced in the hospital wastewater, solid surfaces, and medical devices (Assefa and Amare 2022). It is noteworthy to mention that device-related infections with a biofilm aetiology were the first clinical infections to be recognized (Hall-Stoodley et al. 2004). Several biofilm-related infections such as foreign body-located blood stream infections due to central venous catheter, ventilator-associated pneumonia due to endotracheal tubes, foreign body-located chronic wounds due to soft tissue implants, tissue-located sinusitis due to cystic fibrosis, chronic urinary tract infections due to urinary tract catheterization, and foreign body-located infection due to drainage associated infections have been recognized in the clinical settings (Ciofu et al. 2022). Examples of common pathogens that are involved in the biofilm-related infections are *Escherichia coli, Staphylococcus aureus, Pseudomonas aeruginosa, Enterobacteriaceae, coagulase-negative staphylococci, Acinetobacter *spp*.* and *Enterococcus *spp. (Ciofu et al. 2022). Additionally, the establishment of biofilms will consequently lead to tolerance to both immune system by shielding the embedded microbes even in the presence of both innate and adaptive immune response and tolerance to antimicrobials which necessitates elevated concentrations of antibiotics administered for a longer period, resulting in chronic persistent infections.

Accordingly, the implementation of new antimicrobial strategies to eradicate microbial biofilms as natural products such as phytochemicals and antimicrobial peptides will facilitate the tackling of biofilm-related infections (Quintieri et al. 2022). These natural compounds possess a broad spectrum of activity, are more stable, reliable, and less liable to produce resistance, and may be subjected to chemical modification to achieve better pharmacological and pharmacokinetic properties. Many studies have worked on bioactive compounds from medicinal plants for finding novel natural compounds that act on biofilms with very promising results (Lu et al. 2019; Panda et al. 2020). Unfortunately, not a single FDA-approved drug was manufactured even with this huge work. The solution might be the combination of natural agents together with antibiotics to achieve an inhibitory effect on biofilms (Mishra et al. 2020).

Further investigation is still needed to understand the relationship between biofilms and emerging treatment approaches. As a result, there is a growing interest in studying the ecology of microbial biofilms, especially in the context of their exposure to antibiotics. This review summarizes the recent research on biofilm-associated chronic infections, methods to detect biofilm production, recent approaches to combat biofilms, and future outlook of therapeutic strategy including the development of antimicrobial strategies such as antimicrobial peptides, bacteriophages and biofilm-disrupting agents.

## Biofilms: challenges in antibiotic treatment

**Fig. 1:**
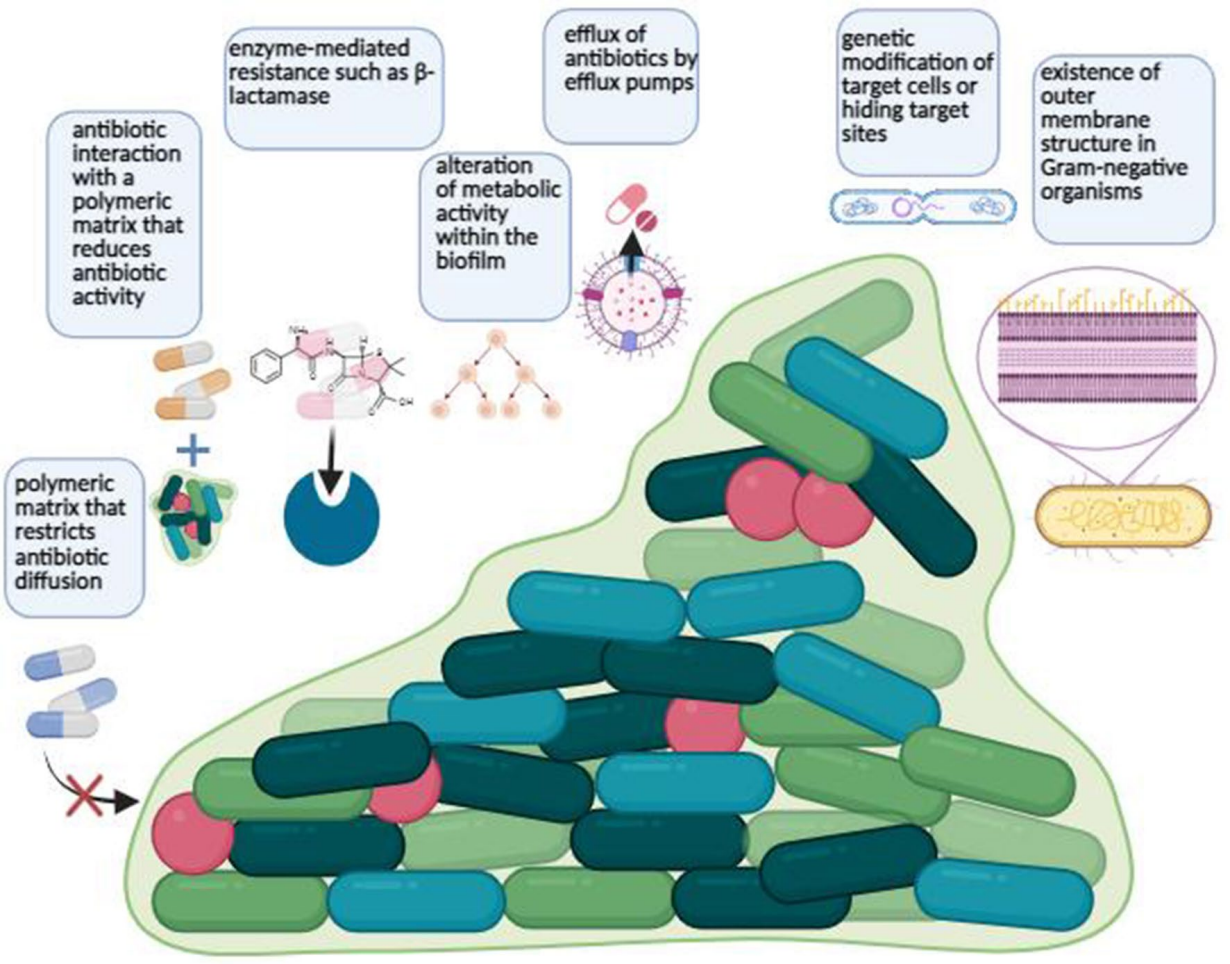
Cartoon representation of biofilm-mediated antibiotic resistance mechanisms

Antimicrobial resistance mechanisms can be categorized into four primary groups: (1) restricting the entry of a drug; (2) modifying the target of a drug; (3) rendering a drug inactive; and (4) actively expelling a drug from the cell. Intrinsic resistance relies on limiting drug uptake, drug inactivation, and drug efflux, while acquired resistance mechanisms may involve drug target modification, drug inactivation, and drug efflux. The specific mechanisms utilized by Gram-negative bacteria and Gram-positive bacteria exhibit variations due to differences in their structures and other factors. Gram-negative bacteria employ all four major resistance mechanisms, whereas Gram-positive bacteria less frequently employ strategies to limit drug uptake (as they lack an outer membrane composed of lipopolysaccharides) and may have limitations in certain types of drug efflux mechanisms (Fig. 1) (Chancey et al. 2012).

It is well known that microbes demonstrating a biofilm phenotype are difficult to manage and their response to antimicrobial therapy is challenging. Consequently, the biofilm development and the resistance to antimicrobial treatment is quietly related (Sharma et al. 2019). The management of microbial resistance is threatened by three main conditions: increase of persistent biofilm-related infections, expansion of antimicrobial resistance and the lack of appropriate therapy (Blanco-Cabra et al. 2021).

It was mentioned that almost 80% of chronic infections in animals and humans are associated with biofilm formation (Sharma et al. 2019). By 2050, the death of 10 million people is expected due to increased rates of morbidity and mortality because of infections caused by MDR pathogens that are the outcome of the misuse of antibiotics together with chronic biofilm-related infections (Inoue 2019). There is significant difference in the susceptibility to antimicrobial therapy of free-living microbes and microbes growing in biofilms. Microbes embedded in a biofilm show higher antibiotic resistance rates than planktonic microbes (Hall and Mah 2017). Importantly, one of the most crucial mechanisms responsible for biofilm recalcitrance to antibiotics is the tolerance of biofilms to antimicrobials. The tolerance of biofilms to antibiotics is coupled to the biofilm’s mode of growth, and if microbes from a biofilm are cultured in planktonic conditions, they will demonstrate susceptibility to the antimicrobial used (Roberts and Stewart 2004).

Therefore, biofilm-related antimicrobial tolerance differs basically from antimicrobial resistance, which can be shown by microbes grown in planktonic culture (Ciofu et al. 2017). This antimicrobial tolerance is attributed to many factors such as restricted penetration of the antibiotic through the biofilm matrix, physiological heterogenicity of microbial cells (it is expected that biofilms hold cells in several states simultaneously: growing, stress adapted, dormant, inactive), expression of biofilm-specific genes in the microbes and the reduced metabolism of the persisters (Ciofu et al. 2017). In addition to all the mentioned mechanisms, in vivo antimicrobial tolerance of biofilms does exist which is much complicated due to the host immune system, access to nutrients and oxygen, and the antibiotics must penetrate different compartments to get the biofilm microbes (Crabbé et al. 2019).

Lately, biofilm was considered as a third compartment, after the tissue (second compartment) and blood (first compartment) that the antibiotics should pass to reach their microbial cells (Cao et al. 2015). The concentration of the local biofilm antibiotic depends on the size and location of the biofilm and on individual drug metabolization. Subinhibitory concentrations of antibiotics at the biofilm site of infection possibly will increase the chance of the occurrence of antibiotic resistance due to selective pressure and increased mutagenesis (Wassermann et al. 2016). Therefore, longer periods of antibiotic therapy together with increasing dosages of antibiotic combinations are highly recommended (Crabbé et al. 2019).

The increased activation of antibiotic resistance mechanisms in biofilms when exposed to antibiotics, as well as their decreased activity in the absence of antibiotic molecules due to metabolism and elimination, plays a significant role in the persistent resistance of biofilms to antibiotic therapy (Ciofu et al. 2017). All the above-mentioned factors related to tolerance of biofilm to antibiotic treatment play a role in the rise of antibiotic-resistant mutants in the normal microbial flora and at the site of infection of the biofilm (Giwercman et al. 1990; Gustafsson et al. 2003).

## Quorum sensing and biofilm formation

Microbial attachment to both living organisms and inanimate surfaces has become a growing concern in our daily lives (Drenkard 2003). The quorum sensing signalling is vital at different stages of biofilm growth, encompassing initiation, formation of the matrix, maturation and detachment. It also has an impact on collective behaviours that shape the structural properties of biofilms, such as surface movement, as well as the synthesis of exopolysaccharides (EPSs) and other adhesive molecules (Hooshdar et al. 2020). As a result, diagnosing and treating biofilm infections can be challenging, often necessitating complex multidrug treatment approaches that frequently prove ineffective in resolving the infection. Targeting individual cells and their quorum sensing (QS) mechanisms has emerged as a highly promising approach for discovering innovative and effective strategies against biofilms (Thabit et al. 2022a, b; Cavalu et al. 2022; Elfaky et al. 2023).

## Microbial biofilms associated with chronic infections

Chronic infections progress more slowly than acute infections, and they frequently have ambiguous signs. With antibiotics, they are extremely challenging to treat. An acquired inflammatory response, which is predominately composed of IgG antibodies and mononuclear leucocytes, is typically what distinguishes chronic inflammation. A persistent inflammatory response and ongoing recruitment of polymorphonuclear leucocytes are features of the inflammatory response in several chronic infections (PMNs). Before the discovery of antibiotics, the most common chronic illnesses were leprosy and tuberculosis, which steadily deteriorated the tissue and damaged the organs (such as the lungs) of patients before causing death (William Costerton 2007).

Patients with illnesses or disorders that impair the principal protective barriers are susceptible to developing chronic infections (innate immunity). The inflammatory anatomical and physiological barriers, such as the skin, mucous membranes and cilia, as well as phagocytic abnormalities, are all affected by this (e.g. PMNs and macrophages). (And et al. 1987; Anwar et al. 1989; Costerton et al. 1999; Donlan 2002; Zimmerli and Trampuz 2011).

### Chronic wounds

Diabetes and cardiovascular disorders have also increased in tandem with the global rise in obesity. These individuals are especially vulnerable to developing chronic wounds, which may host a variety of microbial species (Davies et al. 2004; Gjødsbøl et al. 2006; James et al. 2008; Dowd et al. 2008). Numerous microbial species are found in the deep dermal tissues of all chronic wounds, according to many reported studies (Gjødsbøl et al. 2006; Dowd et al. 2008; Price et al. 2009; Frankel et al. 2009; Thomsen et al. 2010). *S. aureus* is the most frequent bacterium discovered in wounds, while *P. aeruginosa* was found in more than half of the chronic wounds examined (V) (Gjødsbøl et al. 2006; Dowd et al. 2008; Price et al. 2009; Frankel et al. 2009; Thomsen et al. 2010). The area of *P. aeruginosa*-infected wounds was likewise noticeably bigger than that of uninfected wounds, and *P. aeruginosa* similarly appeared to slow or perhaps impede the healing process (Halbert et al. 1992; Madsen et al. 1996; Høgsberg et al. 2011).

According to estimates, 1% and 2% of the populations in Denmark and the USA, respectively, have a wound that is not healing (Gottrup 2004). As a result, chronic wounds are a burden on the healthcare system, and patients who have them suffer, lose their jobs and have a lower quality of life.

### Cystic fibrosis

At the Copenhagen CF Centre, rigorous treatment with high antibiotic doses has been used successfully since 1976 to treat CF patients with persistent *P. aeruginosa* lung infection. Initially, 14 days of routine intravenous anti-*P. aeruginosa* therapy was administered every third month to accomplish this. Daily antibiotic inhalation has been added since 1987. Before 1976, only 50% of CF patients would make it through a persistent *P. aeruginosa* lung infection of 5 years. Most CF patients today have chronic *P. aeruginosa* infections and live for decades (Burmølle et al. 2010).

Chronic *P. aeruginosa* infections require aggressive therapy, yet the germs still exist. The intense treatment delays and lessens the harm that the persistent infection causes, but cannot completely cure it. The lung tissue continues to deteriorate in CF patients with a persistent *P. aeruginosa* infection. Both the infection and the inflammatory processes contribute to the development of this. Lung function deterioration, which is CF patients' leading cause of death, is the result. It is thought that while the Copenhagen CF Centre's current aggressive antibiotic therapy for chronic *P. aeruginosa* infections confines microorganisms to the conductive zone, but does not completely eradicate them. The remaining healthy respiratory zone seems to have long been shielded from severe biofilm infection. This clearly shows that the conductive zone functions as a microbial reservoir, with the microbes structured in mucoid biofilms within the mucus and shielded from antibiotics and host defences (Burmølle et al. 2010).

The idea that biofilms grow in the lungs of CF patients is supported by quite a lot of in vivo investigations. Autopsies, endobronchial lung tissue sections, lung abscesses, freshly removed lung sections and sputum from CF patients have all yielded *P. aeruginosa* clusters. *P. aeruginosa* clusters are comparable to the microbial microcolonies that form a biofilm on inanimate surfaces (Høiby et al. 2010). Additionally, anaerobic or microaerophilic conditions are thought to exist on the mucosal surfaces where *P. aeruginosa* strains have been diagnosed.

*P. aeruginosa* thrives in anaerobic mucosal layers of CF patients' lungs and low oxygen settings in general (Hassett et al. 2009).

### Chronic otitis media

Chronic suppurative otitis media (CSOM) refers to a persistent infection in the middle ear characterized by the presence of a perforated tympanic membrane and the secretion of fluid or discharge from the ear lasting for a duration exceeding 2 months, occurring either continuously or periodically (Artono et al. 2020).

The middle ear infection known without tympanostomy tube insertion is characterized by recurring chronic suppuration, followed by silent dry intervals of varied lengths. Patients with chronic otitis media with dry perforations (COM) or those who have had episodes of acute otitis media (AOM) where treatment has failed or has not been started are at risk for developing CSOM. Once CSOM has developed, the condition is frequently difficult to treat and resistant. Polymicrobial aerobic and anaerobic microbes frequently cause CSOM (Swords et al. 2004). *P. aeruginosa*, *E. coli*, *S. aureus*, and other common aerobic pathogenic microbes found in CSOM, such as pneumococci and *Haemophilus influenzae*, are all recognized as potential biofilm makers. In many chronic middle ear infections over the past 10 years, biofilm is morphologically established experimentally and clinically. The first demonstration was done experimentally on chinchillas having chronic otitis media with effusion (COME) in their middle ear (Rayner et al. 1998; Jurcisek et al. 2005; Reid et al. 2009) and later directly on human clinical mucosal surface lining samples from kids with COME and recurring acute otitis media (rAOM) (Post 2001; Chole and Faddis 2002; Hall-Stoodley et al. 2006).

A common middle ear condition in young children is COME. Additionally, biofilm has been discovered in experimentally produced cholesteatomas in gerbils as well as in human cholesteatoma, a different chronic middle ear condition (Saidi et al. 2016). Biofilm is regularly discovered on prostheses and implanted medical equipment, and it was also discovered on a human cochlear implant (Pawlowski et al. 2005; Bakaletz 2007; Bothwell et al. 2016). Consequently, several chronic infectious middle ear illnesses have been linked to biofilm (Brady et al. 2008). The source and connection of these disorders, as well as the potential harmful role of biofilm, still need to be clarified.

Osteomyelitis (Perloff and Palmer 2018), rhinosinusitis (Connell et al. 2016), urinary tract infections (Trampuz and Zimmerli 2008), and all infections related to foreign bodies inserted into the human body are other chronic infections that have been linked to the biofilm phenotype.

### Infective endocarditis

The mortality rate for infective endocarditis in hospitals is over 20%, despite advances in surgical and medical treatment methods (Beynon et al. 2006). Infective endocarditis is notoriously difficult to treat because it frequently remains resistant to high-concentration intravenous antibiotics for extended periods of time. Up to 50% of infective endocarditis cases require surgical interventions to improve cardiac function and control the infections (Yusuf et al. 2014). The pathogenicity of this infection has been attributed to the microbial ability to bind to damaged prosthetic and natural valves as well as other foreign devices, producing colonization.

The ability of microbes to build biofilms is one of the microbial virulence factors linked to infective endocarditis that is crucially important. There is evidence to support the idea that the infectious colonization that many microbes create on cardiac surfaces to cause infective endocarditis are in fact massive biofilms (Elgharably et al. 2016; Polewczyk et al. 2017).

The following table (Table 1) summarizes some important studies of biofilm associated with chronic infections.

**Table 1:** Studies of biofilm associated with chronic infections

Chronic infections	Study	Refs.
Chronic wounds	A study done by Kwiecinski et al. has demonstrated that the clinical signs of microbial biofilm that colonize wounds, such as a pale wound bed, yellow exudate, necrotic tissue, and clear tissue fluid, are comparable to those of chronic infection wounds	Kwiecinski et al. (2015), Schultz et al. (2017)
	The clinical diagnostic criteria for a biofilm infection were outlined in a World Biofilm Seminar report in 2012 as follows: (1) a pale and edematous wound bed; (2) fragile granulation tissue; (3) a significant amount of yellow exudate; (4) necrotic and rotting tissue; (5) wound pain; and (6) a pungent odour. This criterion was updated in 2017 to include: (1) resistance to treatment with antibiotics or antiseptics; (2) treatment failure	
Cystic fibrosis	According to multiple studies, people with CF may acquire *P. aeruginosa* after visiting hospitals or CF clinics	Römling et al. (1994), Worlitzsch et al. (2002), Campana et al. (2004)
	Römling et al. reported that current antibiotic regimens are unable to completely eliminate *P. aeruginosa* infections in CF airways once they have been established. Chronic *P. aeruginosa* infections are linked to CF patients' clinical state deteriorating and their prognosis getting worse	
	According to Worlitzsch et al.’s study, the hypersecretion of a viscous mucus layer in the CF airway, which creates a low oxygen environment, and the presence of DNA and actin in the CF airway as a result of the necrosis of neutrophils that are recruited into the CF lung as part of the innate immune response are thought to facilitate *P. aeruginosa* ability to grow and establish drug-resistant biofilms in the lungs of CF patients	
Chronic otitis media	Rayner et al. were the first to show a connection between otitis media and biofilm	Rayner et al. (1998), Chole and Faddis (2002), Dohar et al. (2005), Homøe et al. (2009), Lee et al. (2009)
	Dohar et al. first reported chronic otitis media with perforated tympanic membrane using a nonhuman primate model. After the tympanic membrane was punctured and a biofilm-forming strain of *P. aeruginosa* was injected into the middle ear of cynomolgus monkeys, biofilm formation was successfully established in this animal model	
	Chole and Faddis reported that 67 and 95%, respectively, of human cholesteatoma specimens and experimental cholesteatoma from gerbils showed biofilm development frequency	
	Nine out of ten adult chronic otitis media cases and five out of six paediatric chronic otitis media cases that Home et al. described in 2009 had biofilm formation	
	Lee et al. investigated the development of biofilms in ten chronic otitis media patients and ten controls and discovered that biofilms were present in 60% of the study group and 10% of the control group	
Infective endocarditis	Gram staining, culture, and histopathology were used in a comprehensive retrospective investigation on 506 heart valves removed from patients with infective endocarditis, and most of the samples had microscopic positive Gram staining of visible microbes. More than 60% of the valves were microscopically positive even after finishing antibiotic treatment; this number rose to 88% if less than 25% of the course of treatment was completed	ANGRIST et al. (1960), Marrie et al. (1987), Morris et al. (2003)
	The microbes were found to be arranged in colonization as biofilm-like microcolony aggregates, according to histopathological examinations of surgically removed heart valves from infected endocarditis patients as well as heart valves from experimentally infected endocarditis animal investigations according to a study done by Angrist et al.	
	Numerous microbes were found in locations where the surface of the implant was damaged in a study on the ultrastructure of six aortic valves. Even with poor culture results, transmission electron microscopy showed that the microbes were immersed in an electron dense matrix, and that the cell wall and cell division were aberrant	

## Detection of biofilm formation

### Phenotypic detection of biofilm formation

**Fig. 2:**
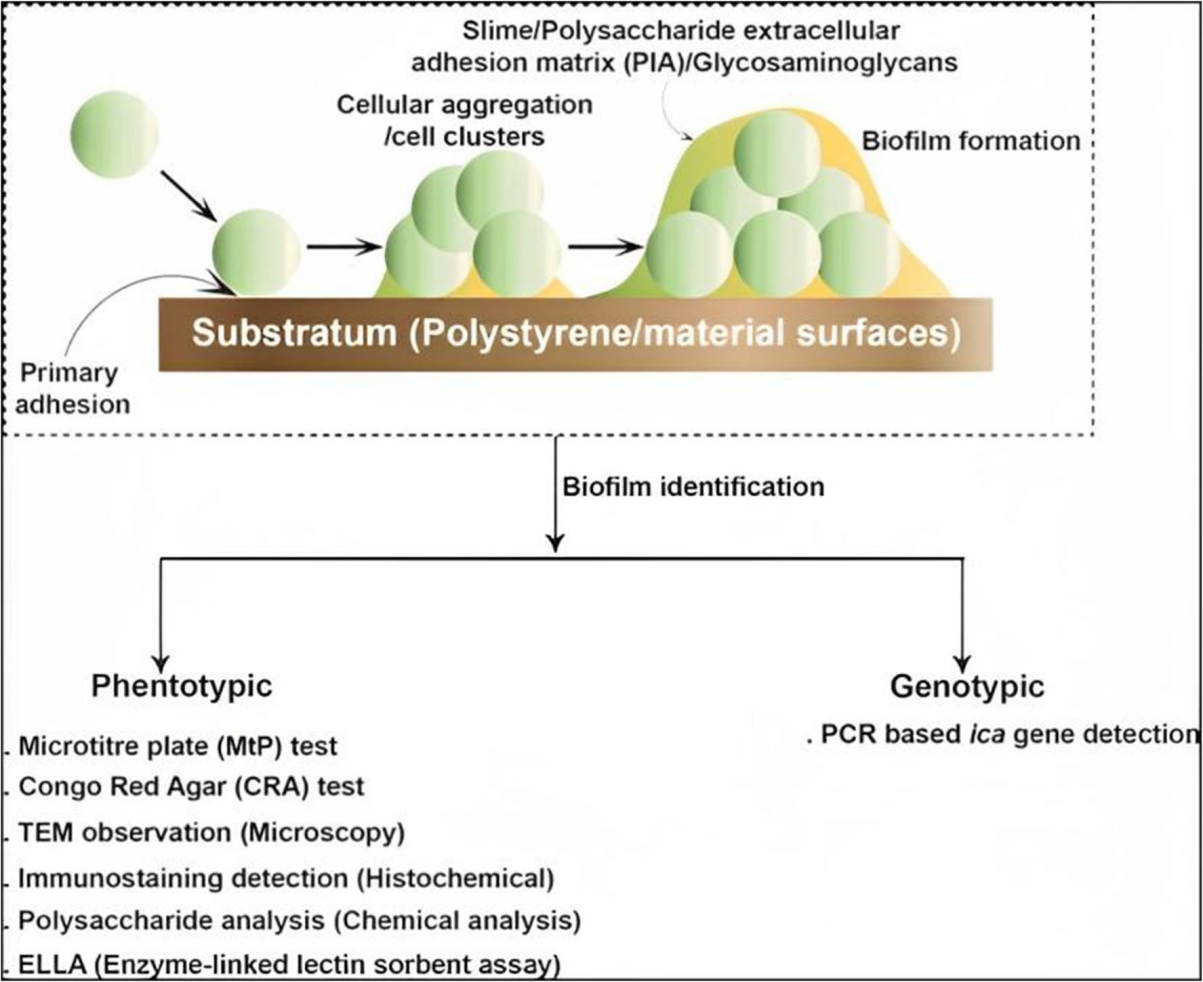
Schematic representation of the biofilm formation and their various detection techniques (phenotypic and genotypic)

Biofilm formation is considered to be one of the major batteries of saprophytic microbiota to become opportunistic pathogen (Kloos and Bannerman 1994; Mertens and Ghebremedhin 2013; Manandhar et al. 2021). Some of the bacteria, such as coagulase-negative staphylococci (CNS) (*Staphylococcus epidermis* and *S*. *aureus*) possess the ability to aggregate and form biofilms by virtue of their secreted mucoid extracellular polymeric substance called polysaccharide intercellular adhesion (PIA) matrix encoded by the *icaA*, *icaD icaB*, and *icaC* genes (Arciola et al. 2014; Manandhar et al. 2021). On the other hand, the biofilm-associated protein (*bap*) found in *A. baumannii* is both a cell surface protein and a virulence factor. It is a large protein (854 kDa) that shares similarities with proteins found in *Staphylococcus* bacteria. *Bap* has been extensively studied in other bacterial genera, particularly in those associated with hospital-acquired infections, including *Enterococcus* spp. and *Pseudomonas* spp. The presence of *bap* is crucial for the formation and maturation of biofilms on both living (biotic) and non-living (abiotic) surfaces (Sharon Goh et al. 2013). *Candida albicans* possesses a diverse array of virulence factors and adaptive characteristics that enable it to successfully infect hosts residing in various environments. The virulence characteristics of *C. albicans* arise from its ability to undergo morphological transitions between two primary forms: yeast and hyphae. The yeast form is crucial for clonal expansion, whereas the invasive hyphal form plays a critical role in promoting virulence. Furthermore, the success of *C. albicans* as a pathogen is attributed to various factors, including the presence of adhesins and invasions on its cell surface, the ability to form biofilms, thigmotropism (response to physical contact), secretion of hydrolytic enzymes, rapid adaptation to changes in environmental pH, metabolic versatility, efficient nutrient acquisition systems, and robust stress responses (Sudbery et al. 2004).The detection of these biofilms could be phenotypic or genotypic (Fig. 2).

#### Microtitre plate test

Among the phenotypic techniques, the microtitre plate (MtP) test is one of the quantitative tests, conducted in 1970 on the biofilm, especially caused by CNS. The MtP test involves the inoculation of biofilm-producing microbes in the 96-well microtitre plate containing tryptic soy broth medium. The inoculated broth is incubated for 18 h. Following the incubation, the microbial layer coating the plastic surface of the wells is washed, fixed and stained with crystal violet and alcian blue or safranin-o that binds to the microbial cell and slime (glycosoaminoglycans), respectively. After the excess stain is removed, the spectrophotometric (OD_570 nm_) measurements are performed with different OD thresholds that classifies the strains as non-adherent non-producers of slime (OD ≤ 0.120), low adherent intermediary slime producers (0.120 < OD < 0.240) or strongly adherent slime producers (OD > 0.240) (Christensen et al. 1985; Deighton et al. 2001; Arciola et al. 2014). Notably, the values chosen for intermediary slime producers are based on the statistical analysis and not on phenotypic observation, which could be one of the limitations of this technique.

#### Congo red agar test

The other phenotypic quantitative in vitro chromatic assessment approach, Congo red agar (CRA) test, has been developed as an alternative to MtP test and is also used to screen the biofilm producing microbes. Precisely, the microbial colonies are cultured on the CRA plates and incubated for 24–48 h, followed by the colour determination of the microbial colonies with deep black to almost black and pink to Bordeaux for slime producers and non-producers, respectively (Freeman et al. 1989). In the recent past a six-colour reference scale has been proposed to facilitate the assessment of colony colour classifications (Arciola et al. 2002). However, the molecular mechanisms underpinning the colour formation of the microbes is still in its initial stage.

It could be possible that Congo red dye directly interacts with certain polysaccharides that brings some metabolic changes in the dye to form a secondary product causing colour variation of the colonies. Notably, this technique also allows direct monitoring of the colonies on the plate of the phase-variant microbes, which is evidenced as pink spikes (virulence factor) on the surface of dark colonies (Ziebuhr et al. 1997) and makes this technique unique for research on microbial physiology. Although the incubation time is prolonged, the CRA approach is still considered to be sensitive and easy to perform in comparison to MtP.

#### Electrochemical impedance spectroscopy

Electrochemical impedance spectroscopy (EIS) has emerged as a powerful tool for the label-free and real-time monitoring of biofilm formation and growth. EIS is a non-destructive and label-free technique that measures the impedance response of an electrochemical system to an applied alternating current (AC) signal. By analysing the impedance spectra, valuable information about the physicochemical properties of the system under study, including biofilm formation and growth, can be obtained (Koo et al. 2022).

Several EIS-based biosensors have been developed for the phenotypic detection of microbial biofilms. These biosensors typically consist of an electrode surface modified with biofilm-specific ligands or antibodies, which selectively capture the target microbes or biofilm components. The impedance changes resulting from microbial binding and biofilm formation are then measured and analysed (Naresh and Lee 2021).

#### Isobaric tags for relative and absolute quantitation-based proteomics

iTRAQ (Isobaric Tags for Relative and Absolute Quantitation)-based quantitative proteomics has emerged as a powerful tool for studying the proteome of microbial biofilms. iTRAQ is a mass spectrometry-based technique that enables relative and absolute quantification of proteins in complex samples. It involves labelling peptides from different samples with isobaric tags, which allows multiplexing and simultaneous analysis. The labelled peptides are then combined, digested, and analysed by mass spectrometry for protein identification and quantification (Asma et al. 2022).

The workflow of iTRAQ-based proteomics for biofilm analysis typically involves biofilm sample collection, protein extraction, digestion, iTRAQ labelling, peptide fractionation, mass spectrometry analysis, and data interpretation. Various sample preparation techniques, such as sonication and enzymatic digestion, can be employed to ensure efficient protein extraction and digestion from biofilm samples. iTRAQ-based proteomics has been widely used to investigate the proteome dynamics of microbial biofilms. It enables the identification and quantification of differentially expressed proteins during biofilm formation, maturation, and dispersal stages. This approach has provided insights into the mechanisms underlying biofilm development, interactions with host cells, and antibiotic resistance. Additionally, iTRAQ-based proteomics has been employed to compare the proteomes of biofilm-associated drug-resistant strains with their planktonic counterparts, revealing potential drug targets and resistance mechanisms (Scorza et al. 2008).

#### Antibodies targeting EPS

Antibodies targeting specific EPS components have emerged as valuable tools for the detection and characterization of microbial biofilms. Antibodies are versatile biomolecules that can recognize and bind to specific target molecules with high affinity and specificity. Antibody-based detection methods, such as enzyme-linked immunosorbent assays (ELISAs), immunofluorescence microscopy, and flow cytometry, can be employed for the detection and quantification of biofilms. By targeting specific EPS components, these antibodies allow selective detection of biofilm structures. The EPS matrix of microbial biofilms consist of various components, including polysaccharides, proteins, and DNA. Antibodies can be generated against specific EPS components, such as exopolysaccharides (e.g. alginate, cellulose), adhesive proteins (e.g. lectins, adhesins), and extracellular DNA (eDNA). These antibodies can be used individually or in combination to target different aspects of biofilm structure and function (Flemming et al. 2016).

### Genotypic detection of biofilm formation

On the other hand, the genotypic approach is based on the identification of the genes encoding for the PIA production as indicated above (Gerke et al. 1998; Götz 2002). All the four genes *icaA*, *icaD icaB*, and *icaC* are organized as the intercellular adhesion *icaADBC* operon along with *icaR*, which is the repressor gene. The individual functionalities of these genes have not been deciphered yet, but it has been known that the contrascription of *icaA* and *icaD* is required for the N-acetyl-glucosaminyltransfarese activity for the PIA polysaccharides oligomer (20 amino acids residues) synthesis. Furthermore, *icaC* gene essentially extends the polysaccharide oligomer to long chain PIA (Gerke et al. 1998).

Notably, the function of *icaB* is still at its infancy; however a plausible hypothesis of de-acetylation of the amino sugars of the PIA chain has been cited. The first typing of these gene was carried out by Southern blotting and hybridization. Thereafter, several primer sets were designed to PCR-amplify *icaA*, *icaD* and *icaC* genes on the extracted genomic DNA directly from the microbial colony and found to be effective in identifying the virulent strains such as of *S*. *epidermis* and *S*. *aureus* (Arciola et al. 2014). However, it has been observed that in the phase variant microbes, despite the presence of *ica* genes, a slime-negative phenotype was evidenced. This is possible because although the *ica* genes are responsible for the polysaccharide production, it has been hypothesized that the phenotypic expression and the virulence effect of the polysaccharide is conditioned by certain regulatory genes such as *atlE*, *sarA*, *agrA* and *mecA* that could potentially modulate PIA functions (Mack et al. 2000). Despite all these limitations, the PCR-based approach is more reliable and accurate in comparison to the phenotypic observation. However, it should be noted that both phenotypic and genotypic approaches should be conducted in parallel to obtain the best biofilm detection option (Arciola et al. 2014).

## Natural compounds with biofilm formation inhibitory potential

For centuries, different civilizations have used natural metabolites and herbal treatments to prevent and treat infectious diseases (Lau and Plotkin 2013; Dhama et al. 2018; Anand et al. 2019). Plants, fungi, and marine organisms demonstrated their potential as abundant sources of novel compounds for preventing the formation of biofilms by various microbial strains (Lu et al. 2019). It has been demonstrated that several of these metabolites prevent QS and control the development of biofilm (Artini et al. 2012; Kouidhi et al. 2015; Asfour 2018). Natural metabolites were said to be able to prevent the development of biofilms in numerous ways, including by preventing the synthesis of peptidoglycans and polymer matrix, interrupting the production of extracellular matrix, repressing cell adhesion and attachment, damaging the structure of microbial membranes, and reducing the production of virulence factors. This would prevent the QS network and biofilm formation (Artini et al. 2012; Asfour 2018; Dong et al. 2018).

**Fig. 3:**
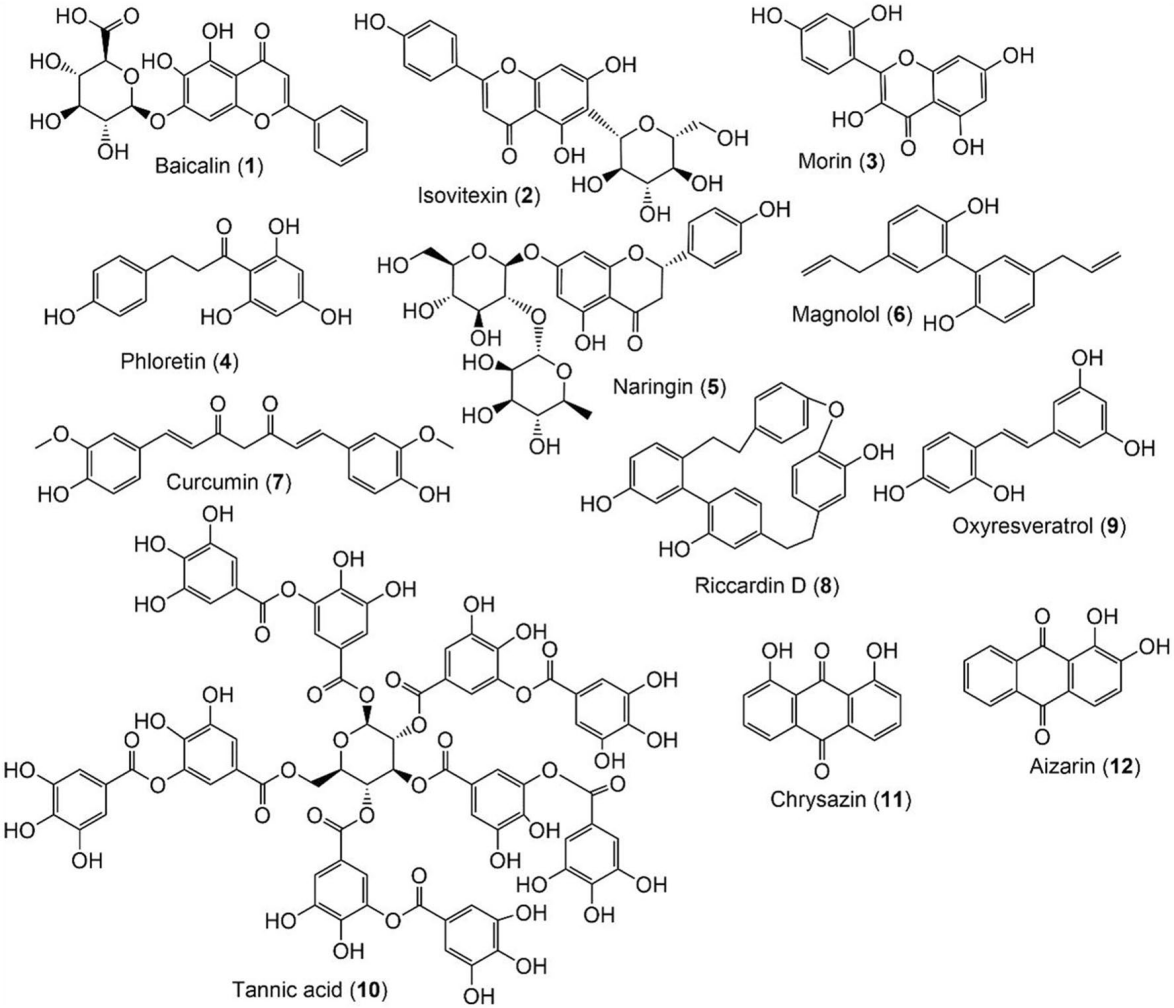
Chemical structures of natural metabolites (**1**–**12**) with biofilm inhibitory potential

**Fig. 4:**
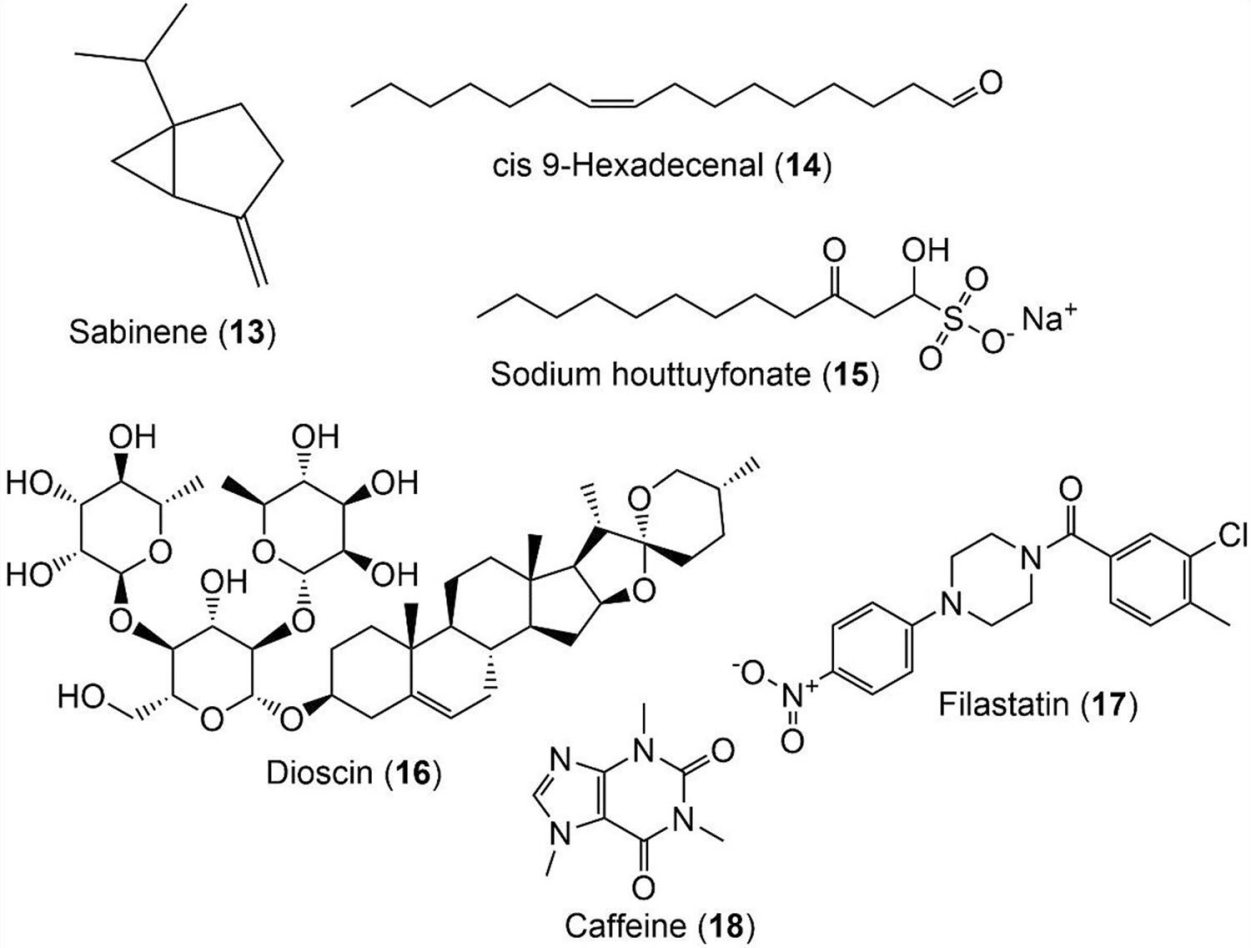
Chemical structures of natural metabolites (**13**–**18**) with biofilm inhibitory potential

Fortunately, clinical and preclinical evaluations of some of them have shown that they have a significant ability to treat or prevent a variety of infectious diseases. It is imperative to create fresh antibiofilm from natural source to increase the microbial resistance resulting from biofilm formation. An overview of a few of the most recent reports on natural biofilm inhibitors was provided in the current review. These metabolites may be used as potent therapeutic agents to increase the effectiveness of antibiotics against biofilm-related illnesses (Figs. 3, 4). Significant antibiofilm potential was found in phenolics, polyacetylenes, terpenoids, alkaloids, lectins, and polypeptides (Yong et al. 2019). Condensed tannins particularly among the phenolics demonstrated antibiofilm activity (Trentin et al. 2011).

In recent years, rising rates of HIV infection, organ transplantation, diabetes, dentures, and the use of anti-cancer, corticosteroid, and broad-spectrum antibiotics have all been linked to rising oral candidiasis occurrence (Chanda et al. 2017). Due to the potential toxicity of clinically used antifungals and the emergence of drug resistance, their treatment is a significant challenge.

### Flavonoids

One of the main flavones isolated from the root of *Scutellaria baicalensis* is called baicalin (**1**) (Zhao et al. 2016). It repressed the QS system by reducing the expression of the rpoS (sigma-S) and H-NS (histone-like nucleoid-structuring) genes through prohibition of AI-2 (autoinducer-2) production. By interfering with the curli-specific genes (csgB and csgA) (Guan et al. 2015), it also interfered with the growth of curli pili and negatively impacted microbial binding and biofilm formation (Peng et al. 2019). In a different investigation, it was found that decreasing the expression of the fimB gene might suppress the synthesis of type 1 pili (Brackman et al. 2009). The Burkholderia cepacian gene CepI increases the production of the QS system-enhancing AHLs (N-acyl-homoserine lactone) signalling molecules C8-HSL (Noctanoyl-homoserine lactone) and C6-HSL (N-hexanoyl-homoserine lactone) by complex formation with their receptors. Baicalin's CepI inhibition suppressed the growth of biofilm by inhibiting the QS system, which prevented the microbial cells from adhering to body surfaces (Slachmuylders et al. 2018).

In addition, the flavonoid-C-glucoside isovitexin (**2**) shows significant Srt-A inhibition (IC50 28.98 µg/mL) capacity, resulting in a decrease in the amount of SpA (staphylococcal-protein A, which was discovered in *S. aureus* 40 years ago, and has the ability to bind to the constant regions of antibodies, making it a valuable tool for extracting immunoglobulins) on the cell surface, indicating the possibility of using it as an anti-*S. aureus* infection agent (Mu et al. 2018). Additionally, it was discovered that the flavonoid, morin (**3**) may be able to inhibit Srt-A in *S. aureus*. Huang et al. showed that it likewise had an inhibitory effect on *S. mutans* Srt-A (IC50 27.2 µM), but had no effect on the survival or expansion of the microbes. At Conc. 30 µM, it partially increased the release of the Pac protein, a cell surface protein antigen c of *S. mutans*, which is determined by the *pac* gene, has been found to show a connection with cellular hydrophobicity and adhesion to tooth surfaces that is not dependent on sucrose, and decreased the mass of the *S. mutans* biofilm without affecting viability. These findings suggested that morin could be valuable as a novel caries-prevention agent (Huang et al. 2009).

The dihydrochalcone, phloretin (**4**), prohibited *L. monocytogenes* biofilm generation (%inhibition 60%, conc. 20 μg/mL), biofilm adhesion, and aggregation, as well as the biofilm thickness as it reduced the agr-system genes by 50% in QS (Wei et al. 2020).

In comparison to tetracycline and ciprofloxacin, the flavanone glycoside naringin (**5**) isolated from grape and citrus fruits showed more effective biofilms influence against *P. aeruginosa*. On catheter surfaces, it was discovered to reduce EPS (extra polymeric substance) biofilm, speed up antimicrobial diffusion, reduce pellicle formation, and reduce microbial flagellar movement. Its combination with antibiotics may be advantageous for newly developed, effective topical antimicrobials as well as for catheter wrapping to fend off infection brought on by biofilms as a result of catheterization (Dey et al. 2020).

### Phenolics

Magnolol (**6**), a previously described polyphenol from the bark of *Magnolia officinalis*, was evaluated in comparison to many oral and common Candida spp. isolates. Magnolol displayed significant antifungal (MICs 16.0–64.0 g/mL) and antibiofilm capability against four tested strains with an average 69.5% inhibition was discovered. Additionally, it resulted in the rupture of plasma membranes and cell walls, the release of intracellular contents, and swelling of the cell walls. It also demonstrated reduced haemolytic activity (%lysis = 11.9%) against red blood cells compared to amphotericin B (%lysis = 25.4%). The molecular docking investigation showed that it interacted with the ergosterol in fungal cell walls to produce its effects (Behbehani et al. 2017). Furthermore, it demonstrated synergism with azoles against *C. albicans* (Sun et al. 2015a) and showed considerable suppression on yeast hyphal transformation, adhesion, and biofilm formation (Sun et al. 2015b). Through pili proteins and/or surface proteins, some pathogenic microbes influence the tissues and cells of the host, playing a crucial role in the infection (Asadi et al. 2018). Staphylococcal species use the sortase A enzyme to adhere surface proteins to their cell walls (Thappeta et al. 2020). Consequently, the binding capacity of *S. aureus* to lgG, fibrinogen, and fibronectin is hindered, thereby diminishing the pathogenicity of the bacterium. The presence of Srt-A also enhances the rate of biofilm formation in certain species of Staphylococci. (Thappeta et al. 2020). In a study conducted by Hu et al. (2013), the potential of curcumin (**7**) to suppress *S. mutans* biofilm was examined. The findings revealed that curcumin effectively inhibited *S. mutans* Srt-A, with an IC50 value of 10.2 µM/L. Moreover, when applied at a concentration of 15 µM/L, curcumin reduced the formation of *S. mutans* biofilm and led to the release of the PAc protein. These results indicate that curcumin exhibits anti-inflammatory effects through a mechanism that hinders bacterial adhesion. (Hu et al. 2013).

### Macrocyclic bisbibenzyls (MBBs)

Riccardin D (**8**), a naturally occurring macrocyclic-bisbibenzyl derivative, was discovered in *Dumortiera hirsuta* and demonstrated in vitro antibiofilm efficacy. Based on XTT (2,3-bis(2-methoxy-4-nitro-5-sulfo-phenyl)-2H-tetrazolium-5-carboxanilide) reduction assay results using CVC (central venous catheter)-infected rabbit model, it has therapeutic and preventative capacity against *C. albicans* biofilm development. This substance inhibited the expression of hypha-related genes, including as *ALS3*, *ALS1*, *EFG1*, *ECE1*, *CDC35*, and *HWP1*, demonstrating that the inhibition of the Ras/cAMP/Efg pathway was the cause of the delayed hypha formation and the defect in biofilm development. Additionally, the fluconazole–riccardin D combination showed increased antifungal potential (Li et al. 2012).

### Stilbenes

Wu et al. evaluated the efficiency of oxyresveratrol (**9**), which was isolated from the heartwood of *Artocarpus lakoocha*, in comparison to *S. mutans* in 2020. The findings showed that this substance (Conc. 250 µg/ mL) decreased bacterial survival rates, hampered the synthesis of H_2_O-insoluble glucans, disrupted the formation of biofilms, and noticeably suppressed the expression of gtfB (glucosyltransferase-I) and gtfC (glucosyltransferase-SI). On the other hand, it increased the expression of ldh and atpD (ATP-synthase subunit-beta) (lactate- dehydrogenase). Additionally, it increased gtfD (glucosyltransferase-S) expression, which aided in the production of glucan that is water soluble. LiaR, vicR, comE, and comD were activated, which improved the self-protective process (Wu et al. 2020). In 2018, Dong et al. demonstrated that tannic acid (**10**), a phenolic molecule, exhibited an exceptional antibiofilm efficiency against *S. aureus* at sub-MIC concentrations by targeting peptidoglycan and causing the integrity of the cell wall to be destroyed. Because of this, it may be a good choice for treating infections brought on by MDR *S. aureus* (Dong et al. 2018). Wu et al. investigation confirmed that it also prevented FabG (β-ketoacyl-ACP-reductase), a crucial enzyme in the production of fatty acids by bacteria (Wu et al. 2010).

### Anthraquinones

Chrysazin (**11**) and alizarin (**12**), according to Manoharan et al. (2017), have antibiofilm capability against *C. albicans* due to a C1 hydroxyl group with toxic influence. Various biofilm-related and hyphal-specific genes are downregulated by it (e.g. ECE1, ALS3, RBT1, and ECE2). Additionally, they (Conc. 2 mg/mL) effectively reduced yeast, to, and hyphal development and increased the *Caenorhabditis elegans* survival rate when infected with *C. albicans* (Manoharan et al. 2017).

In 2019, Jin et al. stated that concanavalin A (*Canavalia ensiformis*`s lectin) had notable antibiofilm effectiveness versus *Listeria monocytogenes* and *E. coli* through its mannose-bound affinity (Jin et al. 2019).

### Monoterpenes

Sabinene (**13**), a component of *Chamaecyparis obtusa* essential oil was reported to repress *Streptococcus mutans* biofilm production and related genes (e.g. *gtfB*, *gtfD*, *gtfC*, *brpA*, *relA*, and *vicR*) expression, suggesting its usage *S. mutans* cariogenic potential inhibitor in oral care products c (Park et al. 2019).

*Aspergillus fumigatus* is a pathogenic fungus that causes a number of serious lung conditions, such as aspergilloma, allergic bronchopulmonary aspergillosis, and invasive pulmonary aspergillosis in people with weakened immune systems who are also hypersensitive to it. It was discovered that this fungus creates a hydrophobic biofilm in the lungs that is made up of several coils of hyphae wrapped in ECM (Tseung and Zhao 2016).

### Fatty aldehydes

*Thuja orientalis*, *Myristica fragrans*, *Cuminum cyminum*, and *Pentaclethra macrophylla* all included the compound cis-9-hexadecenal (**14**). In the broth micro-dilution assay, it prevented 90% of *A. fumigatus* planktonic growth at 0.078 mg/mL (Hoda 2019). In vitro, the checkerboard assay demonstrated that its combination with amphotericin B had increased activity against *A. fumigatus*. In the MTT experiment, it had a 0.156 mg/mL MBEC80 (minimal-biofilm-eradicating concentration-80) vs *A. fumigatus* and scanning electron microscopy revealed the absence of tangled hyphae and ECM in the cis 9-hexadecenal-treated biofilm (Hoda 2019). Additionally, it was harmless to L-132 (a normal human lung epithelial cell line) up to 0.62 mg/mL in the cytotoxicity experiment, suggesting that it could be an exceptional therapeutic agent for disorders linked to *A. fumigatus* (Hoda 2019).

### Sulphonates

Besides sodium houttuyfonate **(15)**, a compound produced by *Houttuynia cordata*, inhibited *P. aeruginosa* motility and biofilm formation while also significantly obstructing the growth of *S. epidermidis* and *C. albicans* (Shao et al. 2012, 2013). It also worked in concert with levofloxacin and Na_2_-EDTA to prevent the formation of biofilms (Huang et al. 2009; Shao et al. 2012). It effectively prevented *P. aeruginosa* biofilm dispersion and BdlA (biofilm dispersion locus A) gene and protein expression, which allowed it to infiltrate *P. aeruginosa* biofilm and suppress the biofilm's life cycle in in vitro research.

### Saponins

Dioscin (**16**), a natural saponin isolated from *Dioscorea panthaica*, suppressed the generation of extracellular phospholipase, the yeast-to-hyphal transition, adhesion to abiotic surfaces, and the formation of biofilms. At high concentrations, it even decreased the viability of preformed biofilms (Yang et al. 2018).

It is noteworthy that several metabolites produced by different actinomycete species demonstrated antibiofilm capability by disrupting cell wall and cell–cell communication (Azman et al. 2019).

### Benzoyl nitrophenylpiperazines

According to Fazly et al., filastatin (**17**) prevented the yeast-to-hyphal transition and hindered the adherence of fungal cells to diverse biomaterials by suppressing HWP1, a hyphal-specific promoter. It's interesting to note that its combination with fluconazole protected *C. elegans* from *C. albicans* infection in vivo. Additionally, it prevented the growth of biofilm in mice with vulvovaginal Candida infection (Fazly et al. 2013).

### Methylxanthines

Additionally, caffeine **(18)** has shown a strong antimicrobial effect against *P. aeruginosa* (MIC 200 µg/mL). Using swarming motility targeting, which was discovered to interact with QS proteins (LasI and LasR), it demonstrated considerable inhibition (Conc. 40 and 80 µg/mL) of *P. aeruginosa* biofilm development and decreased the release of virulence factors. However, it can yet be improved as an antibiofilm agent to manage infections caused by *P. aeruginosa* (Chakraborty et al. 2020).

## Recent advances in biofilm control

Alternative therapies have been sought in an effort to remove or inhibit biofilms because the majority of antibiotics do not work to treat them. From the time the biofilm is formed until it has reached maturity, there are several points where intervention is feasible. Numerous natural substances impair QS, have anti-adhesin activity, stop the formation of films, or have general antimicrobial characteristics. Other treatments involve the use of bacteriophages, viruses that target particular microbes, or enzymes that break down the extracellular biofilm matrix (Sahli et al. 2022).

### Phage therapy

The basis of phage therapy is the use of bacteriophages. The risk of opportunistic infections is decreased because these viruses do not infect humans. Their size typically ranges from 20 to 200 nm, and they are made up of a protein capsid, a typically variable-length tail via which the genetic material is delivered, and fibres at the tail that ensure the host is recognized (pili can be receptors) (Pires et al. 2016).

By utilizing depolymerases, bacteriophages are capable of modifying polysaccharides in biofilms, boosting their penetration into the matrix of the biofilm and hence their effectiveness (Pires et al. 2016). Bacteriophages can also readily move through the biofilm's water channels. Bacteriophages do not have the same antibiotic-like spectrum of action and are specific to certain species population (Sutherland et al. 2004).

Phage-resistant subpopulations can develop within the biofilm community similarly to how they do in planktonic cultures (Fu et al. 2010). After being exposed to anti-*Pseudomonas* bacteriophages, *P. aeruginosa* biofilms develop phage-resistant mutants due to mutations in the genes encoding the phage receptors (Oechslin et al. 2017).

The Soviet Union (USSR) invested in bacteriophages during the twentieth century to treat microbial illnesses. The USSR concentrated on alternative methods of treating its population since it could not afford antibiotics, which are mostly produced by Western nations. Because of this, the Eliava Institute in Georgia, which was formerly a part of the USSR, today boasts one of the largest collections of bacteriophages. The institute began its research on bacteriophages in 1923, just a few years after they were discovered (Oechslin et al. 2017).

Bacteriophages exhibit a high degree of specificity towards their target microbes. They can recognize and infect the specific microbial strains present in biofilms, while leaving beneficial microbes unharmed. This specificity reduces the risk of disrupting the natural microbiota and helps avoid collateral damage to the host. They also have the ability to penetrate the extracellular polymeric matrix that surrounds microbial biofilms. They can target and infect microbes residing deep within the biofilm structure, where antibiotics often struggle to reach. They have the capacity to rapidly evolve and adapt to changes in microbial populations. They can develop new phage variants that overcome microbial resistance mechanisms, including those present in biofilms. This adaptability allows phage therapy to potentially overcome the antibiotic resistance commonly observed in biofilm-associated infections (Furfaro et al. 2018).

While phage therapy holds significant potential for various applications, it is not without its inherent limitations. These limitations include a limited range of hosts, clearance by the immune system, and the emergence of bacterial strains that are resistant to phage treatment (Lin et al. 2022). To advance this rapidly growing industry, it is crucial to address these limitations by implementing well-considered strategies. A comprehensive understanding of phage properties and their interactions with the host is essential to overcome these challenges and drive further development in this field.

### Biofilm-dispersing enzymes

The matrix of the biofilm is a desirable target for antibiofilm therapy because of its porous structure and exposure to the outside environment. The polymers in the biofilm matrix are capable of being broken down by certain enzymes. This stops them from forming, loosens the matrix that has built up on a surface, and makes the microbes in the biofilms more susceptible to antimicrobials (Sahli et al. 2022).

ROCHE laboratory sells Pulmozyme®, a medication, in France as a solution. It is used to treat *P. aeruginosa* infections in people with cystic fibrosis and is based on DNase I. When used against *S. aureus* and *P. aeruginosa*, this medication demonstrates biofilm detachment action (Sahli et al. 2022).

A naturally produced dispersin B by *A. actinomycetemcomitans* which break downs the extracellular polysaccharide poly-(1,6)-*N*-acetylglucosamine (PNAG), which is generated by a variety of microbes, including *S. aureus*, *S. epidermidis* and a variety of Gram-negative Proteobacteria. The breakdown of the biofilm matrix causes it to disperse and makes the remaining microbes more susceptible to an antibiotic's effect (Izano et al. 2008).

The clinical translation of biofilm-dispersing enzymes faces several challenges, including their vulnerability to denaturation, degradation, and clearance when administered in living organisms. To overcome these limitations, drug delivery systems are being developed to encapsulate and protect the enzymes, thereby preserving their enzymatic activity. These systems aim to stabilize the enzymes and shield them from the external environment. Additionally, smart drug delivery systems provide the advantage of targeted specificity, enabling the release of therapeutic payloads specifically at the site of infection while minimizing unnecessary systemic exposure (Thorn et al. 2021).

### Nanotechnology and biofilm prevention

In addition to the traditional approaches for treating microbial biofilms, innovative technologies have also been developed. The design and study of materials at the nanoscale scale, typically between 1 and 1000 nm, can be referred to as nanotechnology. Unique physicochemical and biological features are produced by the nanoscale nature. Their high surface-to-volume ratios, which give properties distinct from those of the bulk, are to blame for this. Nanotechnologies have a wide range of uses, including in the fields of health, energy, military, environment, and information storage (Juang and Bogy 2005; Hussein 2015; Sharon 2019; Khan 2020; Saleem and Zaidi 2020).

In the area of medicine delivery, nanoformulations have various benefits because they allow delivery of medications with low water solubility, safeguarding the medication against enzymatic reactions and medication delivery to the targeted organ, and hence minimizing potential harm and crossing of several membranes that are impervious to conventional drugs; large macromolecules can be delivered intracellularly and transcellularly. Since biofilm pores typically have a width of 50 nm (this value depends on the density of the biofilm), nanomaterials with diameters below this value can easily penetrate the biofilm matrix and get to the microbes that are present in its interior. Drugs that are encapsulated have a distinct biokinetics from those that are free, which concentrates antibiotic action on the biofilm and reduces exposure to human cells. Nanomedicines can decrease doses and toxicity of related medication formulations while also improving the efficacy, specificity, and biodistribution (Peulen and Wilkinson 2011).

Nanomaterial surface functionalization also affects diffusion inside the biofilm. Hydrophobic particles are more evenly distributed within biofilms than hydrophilic ones, and positively charged nanomaterials are better able to enter biofilms with a negatively charged matrix. Additionally, nanoparticles' physical characteristics can be used to combat biofilm. It is possible to employ some inorganic nanomaterials' inherent microbial toxicity or their ability to locally create heat to kill microbes. The concept of a multifunctional nanomaterial combining therapy and diagnosis (theranostics) has evolved in the ongoing quest to enhance therapies while lowering the doses delivered (Li et al. 2014).

Nanoformulations encounter several challenges as they progress from the laboratory to the market. These challenges include the unclear understanding of how nanomedicines interact with biofilms, the complex biological environment within the body, potential toxicity associated with nanomaterials, and limitations in batch production (Thorn et al. 2021). In the future, one potential solution is to utilize more nanocarriers that incorporate biofilm dispersants. This approach can address issues related to the poor stability and hydrophobicity of dispersants, while also potentially mitigating the development of drug resistance.

### Photothermal therapy and photodynamic therapy

Photodynamic therapy (PDT) and photothermal therapy (PTT) have garnered considerable attention and are recognized as successful approaches for treating biofilm infections. PTT enhances the infiltration of antimicrobial agents into biofilms and hampers the emergence of antibiotic resistance. Near-infrared (NIR) light reduces biofilm by approximately 50%, indicating the therapeutic efficacy of heat and precise antimicrobial exposure, with a substantial reduction of up to 90%. However, the high doses of irradiation and concentrations of photosensitizers employed in PTT and PDT to eliminate biofilms can potentially lead to severe tissue damage and inflammation (García et al. 2021; Cai et al. 2021).

Although there is substantial interest in photothermal therapy (PTT), there are still significant challenges that need to be addressed before PTT can be widely implemented in practical applications. One major obstacle is the need for high temperatures (≥ 60 °C) to effectively eliminate bacteria (Huo et al. 2021). However, prolonged exposure to such high temperatures can lead to thermal damage to normal tissues surrounding the bacterial infection sites (Zhu et al. 2016). To overcome this, a more strategic approach is required, involving the optimization of treatment conditions. For instance, shorter treatment times at lower temperatures (around 50 °C) could be explored as a means to achieve effective bacterial eradication while minimizing thermal harm to healthy tissues.

On the other hand, the thickness of human tissue poses a challenge to the application of PDT for antimicrobial infections. This is due to the limited penetration ability or shallow depth of short-wavelength light, or sometimes both factors combined (Gao et al. 2022). To overcome this limitation, the use of longer-wavelength light sources could offer improved tissue penetration, enabling the effective application of PDT in human tissues.

### Laser irradiation

Complete degradation of *S. aureus* and *P. aeruginosa* biofilms is made possible by hybrid metal–polymer nanoparticles (NPs), using laser-induced forward transfer (LIFT). The LIFT method, driven by a pulsed laser beam, deposits a thin layer of an organic or inorganic donor substrate onto a material with high spatial resolution. This transfer might take place in either the solid or liquid phases. *S. aureus* and *P. aeruginosa* biofilms were in contact with a metal NP–polymer composite with LIFT that was constructed of a thin polyethylene terephthalate substrate covered in a layer of silver, copper, or gold metal. These biofilms serve as receptors. The biofilms were not impacted by the laser alone. In contrast, the copper and silver NPs killed them entirely (Nastulyavichus et al. 2020).

### Magnetic disturbance

**Fig. 5:**
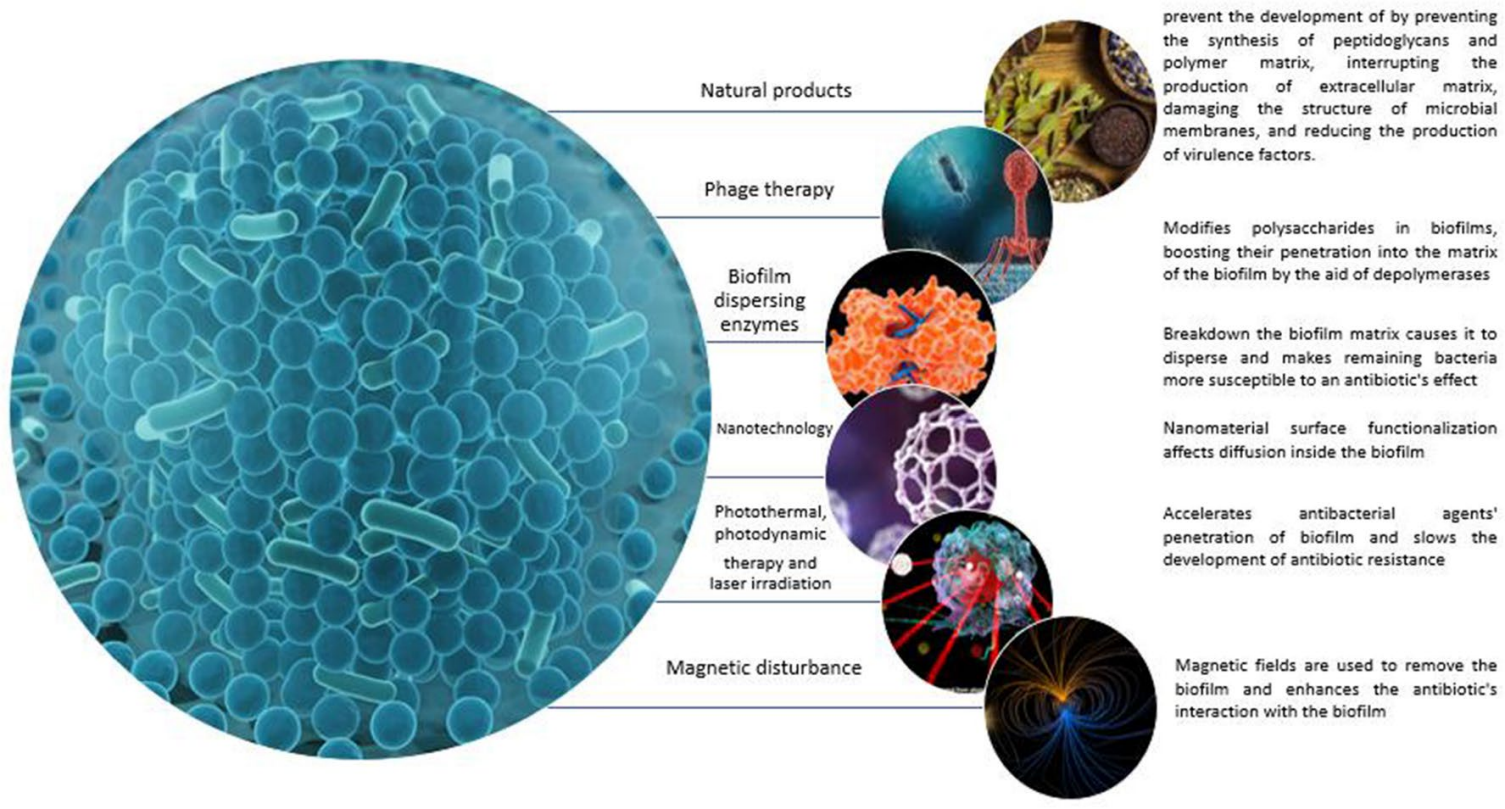
Representation of the advanced control strategies against biofilm and their possible mechanisms

The matrix of methicillin-resistant *S. aureus* (MRSA) biofilms is damaged by IONPs (Fe_3_O_4_ and γ-Fe_2_O_3_) when a magnetic field is applied, according to a recent article (Li et al. 2019). The IONPs are directed and focused at a specific location by the magnetic field. In comparison to 8 nm and 70 nm, 11 nm IONPs exhibit the highest antibiofilm activity, and both the AC and DC magnetic fields that are used to remove the biofilm are more effective than direct contact. IONPs only operate upon the physical breakdown of the biofilm, removing the biofilm from the surface, failing to kill the planktonic MRSA germs. Biofilms can be best dispersed by a spinning DC magnetic field. Due to the IONPs' extended contact with the matrix made possible by the low rotation rate, the biofilm is subjected to strong shearing stresses. Therefore, the IONPs and magnetic field serve as "shield breakers." *S. epidermidis* is penetrated by a nanocarrier called a polymersome that contains IONPS and methicillin. By enhancing the antibiotic's interaction with the biofilm, the IONPs partially deconstruct it, which enhances the synergistic destruction of the sessile population by the antibiotic (Geilich et al. 2017). Magnetic hyperthermia is the process of heating IONPs by applying an AC magnetic field. The separation of the biofilm is caused by local heating caused by IONPs that is substantially greater than that of the medium. The sensitivity of *S. aureus* towards conventional antibiotics is enhanced by mild magnetic NP hyperthermia (Alumutairi et al. 2020). Figure 5 summarizes the advanced control strategies against biofilms.

## Future perspectives

Many studies have worked on bioactive compounds from medicinal plants to find novel natural compounds that act on biofilms, and the results were promising. Unfortunately, not a single FDA-approved drug has been manufactured, even with the considerable amount of research done. Therefore, extended antibiotic therapy and increased antibiotic combinations are highly recommended at the moment, and physicians should be made aware of this.

There are many questions regarding the mechanisms through which microbes maintain a balance between biofilm formation capacity and antibiotic resistance, as well as how resistant strains achieve high levels of biofilm-specific resistance despite producing weak biofilms. Further studies are also needed to determine how the gain of antimicrobial resistance affects biofilm formation. Deeper explorations of plasmid maps and genetic regulation, such as identifying genes involved in biofilm-specific resistance and persisters, would improve our understanding of these processes.

Looking ahead, from our perspectives, there are several promising future strategies for biofilm control, most importantly, combining different treatment modalities, such as PDT, PTT, or other biofilm-targeting strategies, with conventional antibiotics or antimicrobial agents that may prove to be more effective in eradicating biofilms. Synergistic effects between different treatments could enhance biofilm penetration and reduce the risk of antibiotic resistance; developing antibiotics that specifically target biofilms is another potential avenue. These antibiotics would be designed to penetrate biofilm matrices and effectively eradicate the microbes within the biofilm, addressing the challenge of antibiotic tolerance exhibited by biofilm-associated microorganisms.

## Conclusion

The management of microbial resistance is threatened by three main conditions: increased biofilm-related infections, expansion of antimicrobial resistance, and the lack of appropriate therapy. It was mentioned that almost 80% of chronic infections in animals and humans are associated with biofilm formation. Biofilm-related antimicrobial tolerance differs from antimicrobial resistance, shown by microbes grown in planktonic culture.

Alternative therapies have been sought to remove or inhibit biofilms because most antibiotics are not able to treat them. From the time the biofilm is formed until it has reached maturity, there are several points where intervention is feasible. Natural metabolites were able to prevent the development of biofilms in several ways, by preventing the synthesis of peptidoglycans and polymer matrix, interrupting the production of the extracellular matrix, repressing cell adhesion and attachment, damaging the structure of microbial membranes, and reducing the production of virulence factors. A significant antibiofilm potential was found in phenolics, polyacetylenes, terpenoids, alkaloids, lectins, and polypeptides. Condensed tannins, in particular among the phenolics, demonstrated antibiofilm activity.

Some microbes, such as coagulase-negative staphylococci (CNS) (*S. epidermis*) and *S. aureus*, possess the ability to aggregate and form biofilms by their secreted mucoid extracellular polymeric substance called polysaccharide intercellular adhesion (PIA) matrix. Among the phenotypic techniques, the microtitre plate (MtP) test is one of the quantitative tests. Another phenotypic quantitative in vitro chromatic assessment approach, Congo red agar (CRA) test, has been developed as an alternative to the MtP test.

Extended antibiotic therapy and increased antibiotic combinations are highly recommended to deal with biofilm formations. Future studies should focus on the dynamic between biofilm formation and MDR, by, for example, investigating plasmid maps and genetic regulation.

## Data Availability

Not applicable.
